# Bactericidal/Permeability-Increasing Protein Downregulates the Inflammatory Response in In Vivo Models of Arthritis

**DOI:** 10.3390/ijms232113066

**Published:** 2022-10-28

**Authors:** Anna Scanu, Roberto Luisetto, Francesca Oliviero, Francesca Galuppini, Vanni Lazzarin, Gianmaria Pennelli, Stefano Masiero, Leonardo Punzi

**Affiliations:** 1 Rehabilitation Unit, Department of Neuroscience—DNS, University of Padova, 35128 Padova, Italy; 2 Department of Surgery, Oncology and Gastroenterology—DISCOG, University of Padova, 35128 Padova, Italy; 3 Rheumatology Unit, Department of Medicine—DIMED, University of Padova, 35128 Padova, Italy; 4 Surgical Pathology Unit, Department of Medicine—DIMED, University of Padova, 35128 Padova, Italy; 5 Centre for Gout and Metabolic Bone and Joint Diseases, Rheumatology, SS Giovanni and Paolo Hospital, 30122 Venice, Italy

**Keywords:** bactericidal/permeability-increasing protein, arthritis, cytokines, animal models, inflammation, rheumatoid arthritis, crystal-induced arthritis, monosodium urate crystals, calcium pyrophosphate crystals

## Abstract

We investigated the effects of bactericidal/permeability-increasing protein (BPI) alone or in combination with hyaluronic acid (HA) in two animal models: collagen-induced arthritis (CIA) and crystal-induced inflammation. In CIA, mice were intraperitoneally injected with PBS, HA, or BPI plus or minus HA, twice a week for 2 months, and then euthanized to collect paw and blood. Arthritis was assessed in ankle joints by clinical and histological evaluation. Pathogenic crystals were intraperitoneally injected in mice plus or minus BPI, or with a composition of BPI and HA. After sacrifice, total and differential leukocyte counts were determined. Cytokine levels were measured in serum and peritoneal fluids. In CIA mice, BPI improved clinical and histological outcomes (histological scores ≥2-fold), and downregulated inflammatory mediators (47–93%). In crystal-induced inflammation, BPI reduced leukocyte infiltration (total count: ≥60%; polymorphonuclear cells: ≥36%) and inhibited cytokine production (35–74%). In both models, when mice were co-treated with BPI and HA, the improvement of all parameters was greater than that observed after administration of the two substances alone. Results show that BPI attenuates CIA and inflammation in mice, and this effect is enhanced by HA co-administration. Combined use of BPI and HA represents an interesting perspective for new potential treatments in arthritis.

## 1. Introduction

The term arthritis refers to different conditions which can be very different from each other and may affect individuals of all ages, ethnicities, and genders. These disorders are characterized by joint pain accompanied by swelling, stiffness, and inflammation. All these diseases can lead to disability.

Rheumatoid arthritis (RA) and crystal-induced arthritis, including gout and calcium pyrophosphate deposition disease (CPPD), are among the most painful types of arthritis. RA is a systemic autoimmune disorder and is considered the disease prototypical of joint destruction, as synovitis and inflammation of the bone marrow lead to joint erosions [1]. Crystal-induced arthritis results from intra-articular deposition of crystals, such as monosodium urate (MSU) crystals in gout and calcium pyrophosphate crystals (CPP) in CPPD, which induce inflammation and can cause joint destruction [2].

Both patients with RA and crystal-induced arthritis present increased levels of proinflammatory cytokines and chemokines in serum and in synovial fluid (SF). In particular, TNF (tumor necrosis factor)-α, IL (interleukin)-6, and IL-1β have been shown to play a central role in the pathogenesis of these diseases [3,4,5,6].

Currently, treatments for arthritis are aimed at reducing symptoms and inflammation, while preventing permanent joint damage. However, not all patients benefit from the pharmacological treatments available. Moreover, many drugs have potential severe side-effects. Although, in the last decade, the pharmacological therapy for these diseases has significantly improved, there is still a need for new therapeutic targets, along with new effective drugs with fewer side-effects.

Bactericidal/permeability-increasing protein (BPI) is a cationic antibacterial glycoprotein which is produced and secreted in extracellular fluids mainly by polymorphonuclear cells (PMN), but can be also expressed in fibroblasts and epithelial cells [7,8,9]. BPI specifically and selectively acts against Gram-negative infections by damaging bacterial membranes, blunting lipopolysaccharide (LPS), and opsonizing bacteria to increase their phagocytosis by PMN [10,11,12]. Moreover, BPI is not just an antibacterial protein, but it has also been shown to exert additional functions [13]. For instance, it has been shown that BPI acts as an antiangiogenic factor inducing apoptosis in vascular endothelial cells and binding to vascular endothelial growth factor (VEGF) [14,15].

It has been supposed that BPI may play a relevant role in the antimicrobial defense of the lower airways. In agreement with this hypothesis, a high amount of BPI has been detected in the airway surface liquid of patients with acute pneumonia and cystic fibrosis [16]. In addition, different studies reported that dysregulation of BPI in intestinal epithelium is associated with different inflammatory diseases, including Crohn’s disease, ulcerative colitis, and infectious enteritis. High intracellular and extracellular levels of the protein have been identified on homogenates and tissue secretions of biopsy specimens from patients suffering from these diseases [17,18]. Interestingly, high levels of BPI have also been found in SF of patients with RA and reactive arthritis and, to a lesser extent, in osteoarthritis and psoriatic arthritis [19,20]. At present, however, its specific role in these different types of arthropathies has not been determined, and the exact contribution of BPI to innate immunity remains largely unknown.

Considering all of the promising evidence, the possible therapeutic potential of BPI has been examined by different studies. In experimental animal models of infection, administration of recombinant BPI congeners is associated with improved outcomes and a significant increase in survival rates. Clinical studies in humans have demonstrated that treatment with recombinant amino terminal fragments of BPI neutralized endotoxin in patients with acute meningococcal disease [21]. Currently, however, the use of BPI in arthritis or similar conditions has never been evaluated.

Hyaluronic acid (HA) is an anionic non-sulfated glycosaminoglycan widely used for viscosupplementation of joints. It possesses desirable physicochemical properties, including elasticity, high viscosity, biocompatibility, biodegradability, non-immunogenicity, and nontoxicity. Furthermore, studies have stated that HA may also exhibit anti-inflammatory, analgesic, and chondroprotective activities, which may be related to interactions with extracellular matrix (ECM) molecules or specific cell surface receptors, such as CD44, as well as to the ability to reduce the bioavailability of cytokines [22,23,24,25].

The aim of this study was to evaluate the effect of BPI in two mouse models that reproduce certain pathological features of human arthritis: collagen-induced arthritis (CIA) and crystal-induced inflammation. We then investigated whether the addition of HA to the BPI treatment could influence the effect of the protein.

## 2. Results

### 2.1. Effects of BPI Treatment on Collagen-Induced Arthritis

#### 2.1.1. Clinical and Histological Assessments

**Figure 1:**
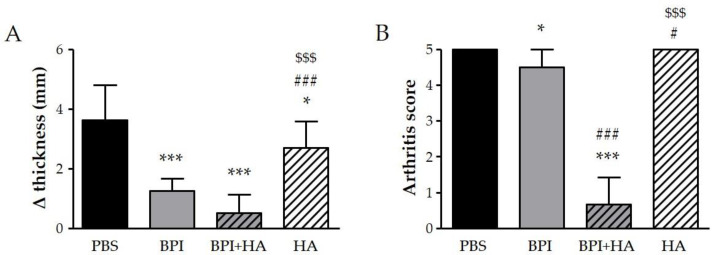
Clinical evaluation of arthritis. Arthritic mice (paw score = 2) were intraperitoneally injected with 200 μL of BPI (50 μg/mL) in PBS in the presence or absence of HA (0.02%) twice a week for 2 months. Mice that received only PBS or HA alone (0.02%) were used as controls. (**A**) The difference between final and initial paw thickness and (**B**) the arthritis score were evaluated at the end of the experiment. Results are presented as the mean ± SD of 12 mice per group. * *p* < 0.05 vs. PBS group, *** *p* < 0.001 vs. PBS group, # *p* < 0.05 vs. BPI group, ### *p* < 0.001 vs. BPI group, $$$ *p* < 0.001 vs. BPI + HA group. BPI: bactericidal/permeability-increasing protein, HA: hyaluronic acid, PBS: phosphate-buffered saline.

We examined the effect of BPI in CIA, which is the most widely used animal model of RA. In our experiment, some mice started to show increased swelling and rigidity on the hind paws, particularly of the ankle joints, 8–10 days after the first immunization, and all mice reached an arthritis score of 2 after 28–33 days. During follow-up, a progressive worsening of arthritis clinical signs, which reached a peak at day 49–56, was observed in mice injected with PBS alone, but not in the BPI-treated group. As reported in Figure 1, at the sacrifice, the difference between the final and the initial ankle thickness, and the Bevaart scores were lower in BPI group than in untreated controls.

**Figure 2:**
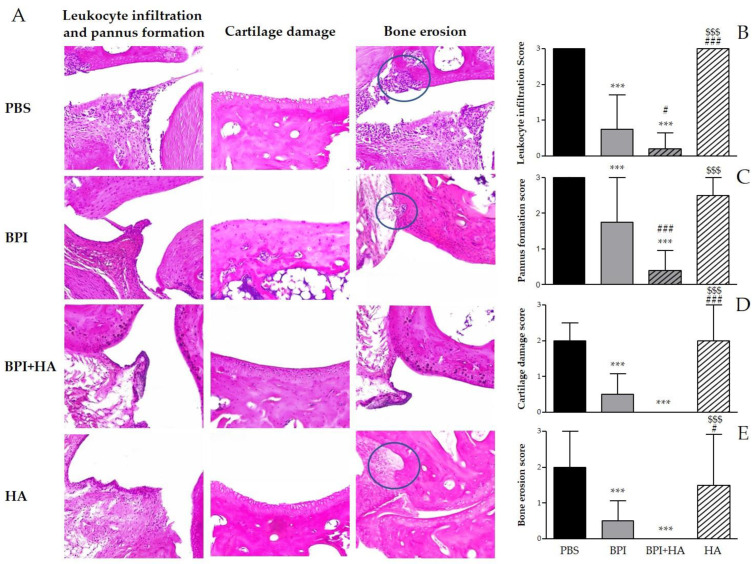
Histological evaluation of arthritis. Histological analysis of the paw tissues was carried out at the end of the experiment. (**A**) Representative hematoxylin and eosin (H&E)-stained sections of the ankle joint of controls and BPI and BPI + HA intraperitoneally injected mice, showing inflammatory leukocyte infiltration, synovial proliferation, cartilage damage, and bone erosion. (**B**–**E**) Histological scores for inflammation, pannus formation, cartilage destruction, and bone resorption. Results are presented as the mean ± SD of 12 mice per group. *** *p* < 0.001 vs. PBS group, # *p* < 0.05 vs. BPI group, ### *p* < 0.001 vs. BPI group, $$$ *p* < 0.001 vs. BPI + HA group. BPI: bactericidal/permeability-increasing protein, HA: hyaluronic acid, PBS: phosphate-buffered saline.

Histological analysis confirmed the development of arthritis and showed the typical features of CIA, including synovial inflammation, leukocyte infiltration, pannus formation, cartilage damage, and bone erosion (Figure 2 and Figure S1). The treatment with BPI improved histological changes as compared to the PBS-injected group. In this experimental setting, BPI decreased histological scores for pannus formation and inflammation two- and fourfold, and decreased histological scores for cartilage and bone destruction fourfold.

Interestingly, co-treatment with BPI and HA led to a significant improvement of clinical symptoms and a reduction in arthritic and histological scores when compared to the PBS group. In addition, the combined use of BPI and HA showed a greater reduction in all parameters evaluated than those observed after administration of BPI alone.

Control mice injected with HA showed only a slight improvement on swelling, bone erosion, and pannus formation as compared with the PBS group.

During experiments, no specific toxicity was observed. Examination of blood smears did not reveal any considerable changes due to toxicity in total and differential white blood cells (data not shown).

#### 2.1.2. Cytokine Serum Levels

**Figure 3:**
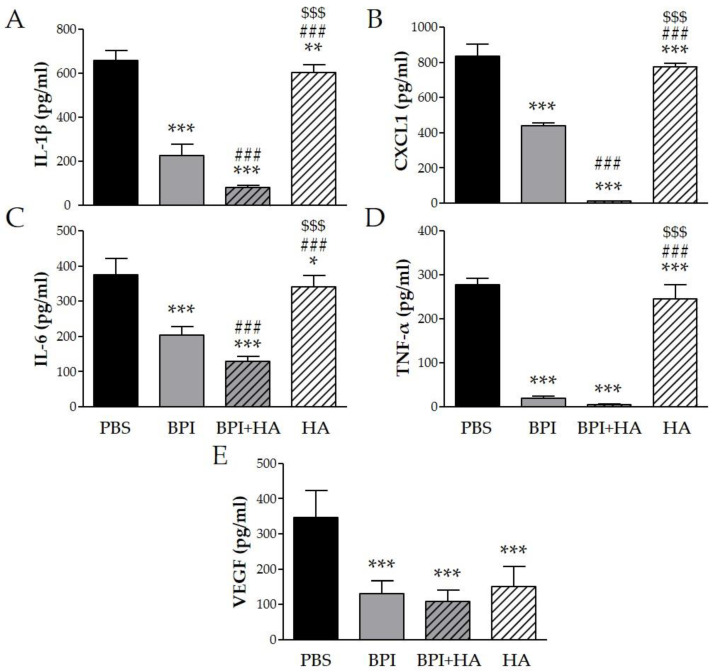
Effect of BPI and BPI + HA treatment on systemic proinflammatory cytokines. Serum from controls and BPI and BPI + HA intraperitoneally injected mice were analyzed for (**A**) IL-1β, (**B**) CXCL1, (**C**) IL-6, (**D**) TNF-α, and (**E**) VEGF by enzyme immunoassay. Results are presented as the mean ± SD of 12 mice per group. * *p* < 0.05 vs. PBS group, ** *p* < 0.01 vs. PBS group, *** *p* < 0.001 vs. PBS group, ### *p* < 0.001 vs. BPI group, $$$ *p* < 0.001 vs. BPI + HA group. BPI: bactericidal/permeability-increasing protein, CXCL1: chemokine (C–X–C motif) ligand 1, HA: hyaluronic acid, IL: interleukin, PBS: phosphate-buffered saline, TNF: tumor necrosis factor, VEGF: vascular endothelial growth factor.

To determine whether the beneficial effect of BPI in the CIA model was mediated through regulation of several inflammatory mediators, we measured the levels of specific cytokines in serum. Arthritis induction led to high levels of IL-1β, CXCL1 (chemokine (C–X–C motif) ligand 1), IL-6, TNF-α, and VEGF, which were markedly decreased after BPI treatment three-, two-, two-, 15-, and threefold, respectively (Figure 3). Furthermore, the lowest levels of the proinflammatory cytokines were detected in serum of mice co-injected with BPI and HA. Indeed, in the latter animals, IL-1β, CXCL1, IL-6, TNF-α, and VEGF concentrations were eight-, 73-, three-, 62-, and threefold lower than in the PBS group. Animals injected with HA alone displayed a reduction in the production of the evaluated cytokines, which remained significantly higher than in BPI groups except for VEGF.

### 2.2. Effects of BPI Treatment on Crystal-Induced Inflammation

**Figure 4:**
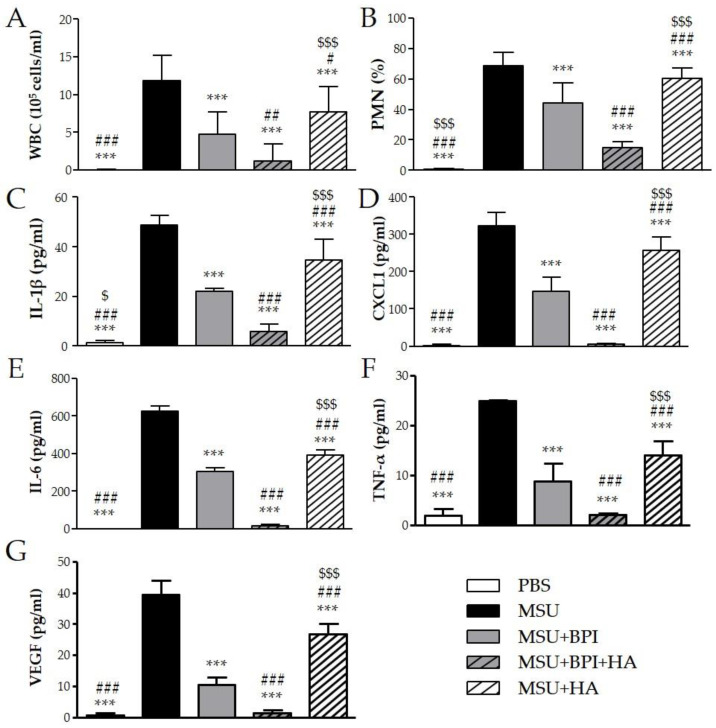
Two milligrams of MSU crystals in 1 mL of PBS were injected into the peritoneum of mice in the presence or absence of BPI (50 μg/mL). In a group of mice, crystals were co-injected with a composition of BPI 50 μg/mL (1.5 μg/g body weight) and HA (0.02%). Controls received the same volume of sterile PBS alone. A group of mice was injected with crystals in the presence of HA (0.02%). Mice were sacrificed after 3 h, and (**A**) the number of leucocytes (white blood cells (WBC)) and (**B**) the percentage of polymorphonuclear cells (PMN) accumulated in peritoneal fluids were determined. Supernatants from the peritoneal wash fluids were analyzed for (**C**) IL-1β, (**D**) CXCL1, (**E**) IL-6, (**F**) TNF-α, and (**G**) VEGF. Results are presented as the mean ± SD of 14 mice per group. *** *p* < 0.001 vs. MSU group, # *p* < 0.05 vs. MSU + BPI group, ## *p* < 0.01 vs. MSU + BPI group, ### *p* < 0.001 vs. MSU + BPI group, $ *p* < 0.05 vs. MSU + BPI + HA group, $$$ *p* < 0.001 vs. MSU + BPI + HA group. BPI: bactericidal/permeability-increasing protein, CXCL1: chemokine (C–X–C motif) ligand 1, HA: hyaluronic acid, IL: interleukin, PBS: phosphate-buffered saline, MSU: monosodium urate crystals, PMN: polymorphonuclear cells, TNF: tumor necrosis factor, VEGF: vascular endothelial growth factor, WBC: white blood cells.

**Figure 5:**
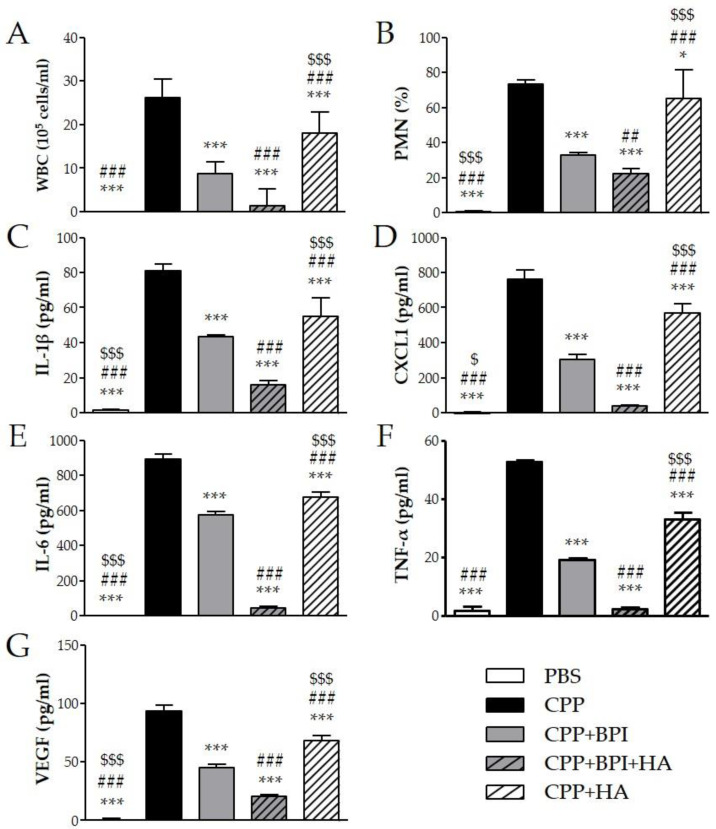
Two milligrams of CPP crystals in 1 mL of PBS were injected into the peritoneum of mice in the presence or absence of BPI (50 μg/mL). In a group of mice, crystals were co-injected with a composition of BPI 50 μg/mL (1.5 μg/g body weight) and HA (0.02%). Controls received the same volume of sterile PBS alone. A group of mice was injected with crystals in the presence of HA (0.02%). Mice were sacrificed after 3 h, and (**A**) the number of leucocytes (white blood cells (WBC)) and (**B**) the percentage of polymorphonuclear cells (PMN) accumulated in peritoneal fluids were determined. Supernatants from the peritoneal wash fluids were analyzed for (**C**) IL-1β, (**D**) CXCL1, (**E**) IL-6, (**F**) TNF-α, and (**G**) VEGF. Results are presented as the mean ± SD of 14 mice per group. * *p* < 0.05 vs. CPP group, *** *p* < 0.001 vs. CPP group, ## *p* < 0.01 vs. CPP + BPI group, ### *p* < 0.001 vs. CPP + BPI group, $ *p* < 0.05 vs. CPP + BPI + HA group, $$$ *p* < 0.001 vs. CPP + BPI + HA group. BPI: bactericidal/permeability-increasing protein, CPP: calcium pyrophosphate crystals, CXCL1: chemokine (C–X–C motif) ligand 1, HA: hyaluronic acid, IL: interleukin, PBS: phosphate-buffered saline, PMN: polymorphonuclear cells, TNF: tumor necrosis factor, VEGF: vascular endothelial growth factor, WBC: white blood cells.

To evaluate the effect of BPI in an acute inflammatory condition, we used the model of peritonitis induced by pathogenic crystals. Injection of suspension of MSU or CPP crystals induced an inflammatory response characterized by leukocyte recruitment, mainly polymorphonuclear cells (PMN), and increased the levels of IL-1β, CXCL1, IL-6, TNF-α, and VEGF in lavage fluids collected. As shown in Figure 4, the administration of BPI together with MSU crystals significantly reduced leukocyte counts and inhibited the release of proinflammatory mediators. Indeed, in the presence of BPI, the white blood cell (WBC) influx and the PMN rate decreased by 60% and 36%, respectively. Concomitantly, BPI inhibited the MSU crystal-induced production of IL-1β, CXCL1, IL-6, TNF-α, and VEGF two-, two-, two-, three-, and fourfold, respectively. Similar results were observed for CPP crystal-induced inflammation (Figure 5). The co-injection of CPP crystals and BPI inhibited leukocyte infiltration by 67% and PMN recruitment by 55%, as well as reduced the concentrations of all inflammatory mediators (twofold for IL-1β, CXCL1, IL-6, and VEGF; threefold for TNF-α). Similarly to the results previously described using the CIA model, all parameters were considered further diminished when HA was added to the treatment. In the latter setting, the cell counts and cytokine production were significantly reduced, not only in comparison with the mice injected with crystals alone, but also in comparison with those co-treated with crystals and BPI. Of note, inflammation induced by the two different types of crystals was significantly reduced also after treatment with HA alone, even though cell counts and cytokine levels remained significantly higher when compared to the treatments where BPI was present. Injection of PBS or BPI alone did not induce any inflammatory effect.

## 3. Discussion

This study demonstrated that BPI attenuated disease progression in CIA mice and reduced MSU- and CPP-induced acute inflammation in the mouse peritonitis model. In CIA model, administration of BPI ameliorated the severity of arthritis and decreased circulating levels of proinflammatory mediators. The injection of MSU or CPP crystals caused a leucocyte infiltration and an increase in IL-1β, CXCL1, IL-6, TNF-α, and VEGF in lavage fluids from mice with peritonitis, which diminished in the presence of BPI. We also demonstrated that the beneficial effect of BPI is greatly enhanced by the co-administration of HA.

CIA is a well-established experimental model that, through an autoimmune reaction to a cartilage component, can lead to a chronic destructive polyarthritis. In this model, mice develop symptoms of joint swelling and deformity, accompanied by destruction of joints and an inflammatory reaction [26,27]. Our results show that, in CIA mice, BPI administration ameliorated disease progression in terms of symptoms and damage to joint components, including bone and cartilage erosion and synovial hyperplasia. Since BPI has been detected in synovial fluid from patients with different arthritis [19], our data suggest that BPI could have a protective effect in joints and that it may act as immune effector even without a direct interaction with microbial cell-surface and/or membrane components.

Moreover, BPI displayed anti-inflammatory activity by reducing articular leukocyte infiltration and circulating levels of proinflammatory cytokines. It is known that cytokines play an important role in modulating immune responses in inflammatory and autoimmune diseases [28,29]. In particular, IL-6, TNF-α, IL-1β, and IL-8 play a key role in the pathogenesis and development of arthritis, and high levels of these cytokines have been detected in both the serum and the SF of RA, gout, and CPPD patients [3,5,30,31,32,33,34]. Similarly, we detected, in the serum of CIA mice, high levels of IL-6, TNF-α, IL-1β, and CXCL1, the murine IL-8 homolog, which were significantly suppressed by BPI.

Interestingly, as in RA patients [35], VEGF concentrations were elevated in the serum of arthritic mice. In this context, different studies have reported that increased VEGF level in the serum of patients with RA correlated with disease activity, radiographic progression, and C-reactive protein concentration [36,37]. Moreover, it has been demonstrated that VEGF is highly expressed in the synovial cells of RA patients and regulates osteoclast differentiation, thus suggesting an important role for this proangiogenic factor in promoting synovial hyperplasia and bone destruction [38]. In our experiments, BPI administration also showed modulatory activity on VEGF production, reducing its levels in CIA mice. This agrees with previous reports demonstrating that BPI inhibits angiogenesis inducing cell apoptosis, inhibiting migration of endothelial cells and complexing the VEGF [14,15,39,40].

In our study, BPI also showed its efficacy in acute inflammatory conditions, such as that promoted by microcrystals. Using the peritonitis model, we determined that BPI exerts an anti-inflammatory effect through the reduction in leukocyte recruitment and downregulation of proinflammatory cytokine production.

Together these results are in agreement with previous studies demonstrating that BPI displays anti-inflammatory properties in both in vitro and in vivo experimental models. For instance, a recent study reported enhanced neutrophil recruitment and inflammatory cytokines in BPI-deficient mice infected with Pseudomonas aeruginosa, which were corrected after administration of exogenous BPI [21]. In addition, it was shown that BPI prevents TNF secretion by human PBMC in response to LPS in a dose-dependent manner [41]. However, although the anti-inflammatory action of BPI is well known, this effect has only been demonstrated in the presence of bacterial infections so far. Indeed, this protein exerts an antimicrobial activity by damaging bacterial membranes, neutralizing endotoxin, and exhibiting opsonic function [9,21]. Otherwise, in our experiments, we used MSU or CPP crystals, which are pathogenic but not infectious agents, thus suggesting for the first time that BPI can also exert an anti-inflammatory activity in sterile inflammation.

We also demonstrated that the combined use of BPI and HA was significantly effective in reducing clinical signs in animals with CIA and inflammation in both experimental models used. In particular, the co-administration of the two agents showed more remarkably reduced paw swelling and arthritic score than their separate use, thus suggesting that the treatment with BPI + HA not only slows the progression of the disease but even reverses it, as demonstrated by a final arthritis score (paw score = 0.67 ± 0.75) lower than that considered at the start of treatment (paw score = 2).

Moreover, BPI + HA as BPI alone reduced the recruitment of leukocytes both in the peritoneal cavity and in the joint of arthritic mice, indicating that the protein can act directly locally, as well as at distant sites from the injection site, and that this effect is not altered by the presence of HA.

Lastly, the lowest levels of CXCL1, TNF-α, IL-6, IL-1β, and VEGF were observed in both the serum and the peritoneum lavage fluid of mice co-injected with BPI and HA. These results confirm the anti-inflammatory effect of HA, already demonstrated in several experimental models of inflammation, including that induced by pathogenic crystals [22,42], and that the two components potentiate the action of each other, thus suggesting a synergistic effect.

Despite these promising results, available data do not allow identifying any specific mechanism of action of the effects of BPI and behind the associations between the two molecules. In addition to its well-known anti-infective properties, it has been also hypothesized that BPI may interact with some membrane receptors, such as Toll-like receptor 4 (TLR4) and glypican 4 [43,44]. The latter hypothesis suggests that, in our experiments, BPI may bind specific receptors in target cells, the affinity of which could be increased by the assumption of a slightly different conformation following the binding with HA. Alternatively, the activity of the protein could be amplified by the direct interaction of HA with cells and extracellular membrane [25].

Following the beneficial effects obtained in both animal models, BPI, strengthened by the combination with HA, may play a potential therapeutic role in arthropathies. A formulation for local intra-articular use, for instance, might find a wide range of applications in rheumatology, particularly in monoarticular joint diseases, which do not require systemic pharmacological approach.

## 4. Materials and Methods

### 4.1. Animals

Male C57/Bl6 mice were provided by the breeding facility of the Interdepartmental Research Center of Experimental Surgery of the University of Padova (Breeding Authorization number 102.2004-A issued from National Health Ministry under Italian Law DLGS 16/2014) and maintained on standard laboratory diet and water ad libitum. All experimental procedures were approved by the Padova University Animal Ethic Committee and Italian Ministry of Health (Rome, Italy) registered under #1105/2016-PR and #602/2017-PR.

### 4.2. Murine Model of Collagen-Induced Arthritis (CIA)

#### 4.2.1. Arthritis Induction

**Figure 6:**
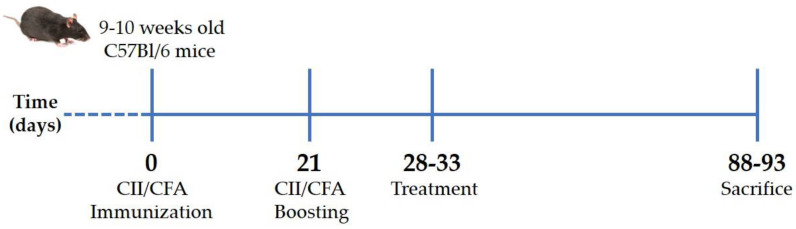
Experimental scheme of collagen-induced arthritis. Arthritis was induced by intradermal injection at the base of the tail of type II collagen and complete Freund’s adjuvant mixture. After 3 weeks (21 days), the initial immunization mice received a booster dose. When mice reached an arthritis score of 2 (28–33 days), they received treatments twice a week for 2 months and were then euthanized. CII: type II collagen, CFA: complete Freund’s adjuvant.

CIA was induced in 48 C57Bl/6 mice aged 9–10 weeks, with a mean body weight of 35.61 ± 1.14 g, by immunization with type II collagen (CII; code: 804001, MD Biosciences GmbH, Zurich, Switzerland)/complete Freund’s adjuvant (CFA; code: F5881, Merck Life Science S.r.l., Milano, Italy) emulsion at days 0 and 21 [45]. Briefly, anesthetized mice were injected intradermally at the base of the tail with 2 mg/mL CII emulsified 1:1 with 1 mg/mL CFA (70 μL; T = 0). After 3 weeks (T = 21), the initial immunized mice received a booster dose (Figure 6). Development and severity of arthritis were monitored by measuring paw swelling with a digital caliper (Kroeplin, Schlüchtern, Germany) and scored from 0 to 5 according to the Bevaart clinical scoring system [45] every other day. Animal weight was also recorded.

#### 4.2.2. Treatment

All mice showed signs of arthritis that reached a severity score of two between the 28th and 33rd day after immunization. At this point, mice were randomized into four groups (*n* = 12 per condition), which received intraperitoneal injection with 200 μL of (i) BPI (code: 16-14-021609-100UG, Athens Research & Technology, Inc., Athens, GA, USA) (50 μg/mL) in PBS, (ii) BPI in the presence of HA (0.02%) (BPI+HA), (iii) only PBS, or (iv) HA alone (0.02%; molecular weight 500–730 KDa). Treatment and arthritis evaluation were carried out twice a week for 2 months. Arthritis severity was assessed as described above. At the experiment endpoints, mice were euthanized by an excess of anesthesia (inhalation of sevoflurane at 8%), and pathological examinations were also performed (Figure 6).

#### 4.2.3. White Blood Cell Counts, Serum Cytokine Determination, and Histological Evaluation

At the sacrifice, blood was collected by cardiac puncture. A small quantity of the blood was used to prepare blood smears in order to evaluate the total and differential white blood cell count using May–Grunwald–Giemsa staining. The remaining blood was centrifuged at 3000 rpm for 10 min to obtain serum, and the levels of IL-1β (code: 88-7013-88, eBioscience, San Diego, CA, USA), IL-6, TNF-α (codes: 431301 and 430904, BioLegend, San Diego, CA, USA), CXCL1 (code: 900-K127, Peprotech, Rocky Hill, NJ, USA), and VEGF (code: DY493-05, R&D Systems, Inc., Minneapolis, MN, USA) were measured by sandwich ELISA. Hind paw specimens were fixed in formalin, embedded in paraffin, and stained with hematoxylin and eosin for histological analysis. Paws were assessed for cartilage destruction, pannus formation, bone erosion, and inflammatory changes according to the number of inflammatory cells in ankle joints. The severity of each parameter was scored separately on a scale from 0 to 3 (0 = normal, 1 = mild effect, 2 = moderate effect, 3 = severe effect) [46].

### 4.3. In Vivo Model of Crystal-Induced Inflammation

Inflammation was induced by injection of MSU or CPP crystals, commonly involved in gout and CPP crystal-induced arthritis, respectively. All animals used for the experiments were male aged 8–9 weeks and a mean of the body weight of 34.5 ± 2.03 g (*n* = 14 per condition).

#### 4.3.1. Preparation of Monosodium Urate and Calcium Pyrophosphate Crystals

MSU and CPP crystals were prepared using Denko’s and Cheng’s method [47,48]. Their shape and birefringence were assessed by compensated polarized light microscopy. Crystals were sterilized by heating at 180 °C for 2 h before each experiment. Less than 0.015 5 EU/mL endotoxin was measured in crystal preparations using a Limulus amoebocyte lysate assay (code: E8029, Merck Life Science S.r.l., Milano, Italy).

#### 4.3.2. Induction of Inflammation and Treatment

Two milligrams of MSU or CPP crystals in 1 mL of phosphate-buffered saline (PBS) were injected into the peritoneum of mice according to the method described by Leypoldt et al. [49,50] in the presence or absence of BPI (50 μg/mL). In a group of mice, crystals were co-injected with a composition of BPI 50 μg/mL (1.5 μg/g body weight) and HA (0.02%; molecular weight 500–730 KDa). Controls received the same volume of sterile PBS alone. A group of mice was injected with crystals in the presence of HA (0.02%). Mice were sacrificed after 3 h by an excess of anesthesia (inhalation of sevoflurane at 8%), and peritoneal fluids were harvested by washing with 2 mL of PBS. Immediately after, lavage fluids, collected with eventually present exudate, were assessed for total and differential WBC count to determine the inflammation degree. The fluids were then centrifuged at 3000 rpm at 4 °C for 10 min, and the supernatants were removed to measure the concentration of IL -1β, IL-6, CXCL1, TNF-α, and VEGF by sandwich ELISA [51].

### 4.4. Statistical Analysis

Data are reported as the mean ± SD. Statistical differences between experimental groups were assessed by nonparametric one-way analysis of variance (ANOVA), as the variables were not normally distributed (Shapiro–Wilk normality test). Multiple comparisons were performed by Dunnett’s test. GraphPad Prism software 5.0 (Version 5.01, GraphPad Software, Inc., San Diego, CA, USA) was used for analysis, and a *p*-value < 0.05 was considered significant.

## 5. Conclusions

In conclusion, this study demonstrated that BPI attenuates progression of CIA in mice and downregulates the inflammatory process in crystal-induced inflammation and CIA. These effects are greatly enhanced by co-administration of HA. Our results strengthen the role of BPI as a modulator of inflammation by demonstrating its beneficial effect even in the presence of nonbacterial inflammatory conditions. The use of BPI alone or combined with HA represents an interesting strategy for developing new potential interventions in the treatment of arthritis. Further studies should identify the exact mechanism via which BPI exerts this anti-inflammatory effect.

## 6. Patents

Patents resulting from the work reported in this manuscript have been filed in Italy (application numbers: 102017000108102 and 102018000003756) and PCT (application number: PCT/EP2018/074889).

## Data Availability

The data presented in this study are available on request from the corresponding author.

## Supplementary Materials

The following supporting information can be downloaded at https://www.mdpi.com/article/10.3390/ijms232113066/s1.
