# Supplementary material for: Bactericidal/Permeability-Increasing Protein Downregulates the Inflammatory Response in In Vivo Models of Arthritis
Source: Int J Mol Sci. 2022 Oct 28;23(21):13066. doi: 10.3390/ijms232113066 (PMC9656099; doi:10.3390/ijms232113066)
Supplement: Supplementary file 1 [file ijms-23-13066-s001.zip › ijms-1962727-supplementary.pdf]

International Journal of *Molecular Sciences*

MDPI

Supplementary Figure S1

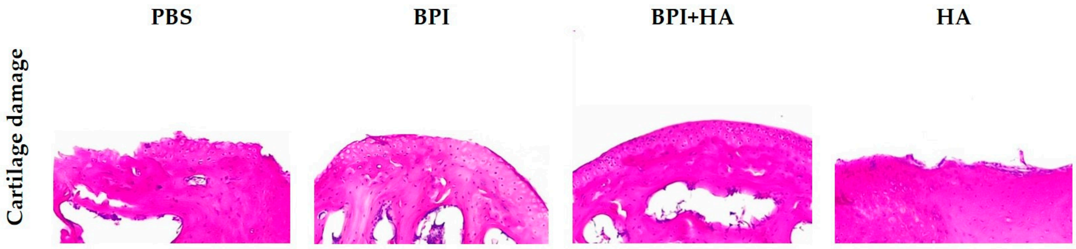

Figure S1. Representative hematoxylin and eosin (H&E)-stained sections of the subtalar joint of controls, BPI, and BPI + HA intraperitoneally injected mice showing cartilage damage. BPI: bactericidal/permeability-increasing protein, HA: hyaluronic acid, PBS: phosphate-buffered saline.
